# Medical Honey in Head and Neck Cancer

**DOI:** 10.7759/cureus.52822

**Published:** 2024-01-23

**Authors:** Joel Badders, Orly Coblens, Viran Ranasinghe, Sepehr Shabani

**Affiliations:** 1 School of Medicine, University of Texas Medical Branch, Galveston, USA; 2 Otolaryngology, University of Texas Medical Branch, Galveston, USA

**Keywords:** postoperative wounds, ­wound healing, radiation-induced mucositis, head and neck neoplasms, medical honey

## Abstract

Medical honey has been recognized for its medicinal properties for thousands of years, and several medical honey products have entered the market over the last two decades. In vitro studies have shown that honey has antimicrobial properties, protects against bacterial growth, and improves wound healing. However, these products are not widely used for wound treatment in head and neck surgery. Additionally, honey has been utilized in those undergoing radiotherapy for head and neck cancer (HNC) to treat radiation-induced mucositis. This literature review aims to describe and assess the utility of medical honey for patients undergoing treatment for HNC and thus review publications on medical honey for treating postoperative wounds and preventing radiation-induced mucositis.

Ovid (Medicine), PubMed (Medline), and Google Scholar were searched using keywords related to medical honey and HNC. All abstracts retrieved in the search were screened for content relevance. Three randomized controlled trials on wound healing were reviewed and assigned a score using the revised Cochrane risk of bias tool for methodological quality. Three meta-analyses assessing radiation-induced mucositis were reviewed and evaluated.

Using medical honey on postoperative wounds was associated with shorter hospital stays, faster healing of palatal graft defects, and reduced pigmentation of thyroidectomy scars. Medical honey had no impact on bacterial culture rates or other aesthetic measures. For patients undergoing radiation for HNC, orally ingested honey was associated with less weight loss and delayed the onset of severe radiation-induced mucositis. However, results across different meta-analyses were mixed.

Minimal evidence supporting the use of honey in postoperative wounds for patients with HNC exists. However, the studies reviewed here, combined with in-vitro studies and studies in other anatomical regions, show honey may offer some wound healing benefits. More robust studies are needed to confirm the potential benefits of medical honey in the postoperative wounds of HNC patients. While radiation-induced mucositis continues to be a debilitating adverse effect of HNC treatment, the literature reviewed supports honey as a safe complementary therapy in preventing radiation-induced mucositis.

## Introduction and background

Cancers of the head and neck continue to have a significant global burden of disease, with a typical yearly incidence ranging from 500,000 to over 1,000,000 cases worldwide, as well as a substantial economic burden [1-3]. More widespread global use of multiple treatment modalities including surgery, radiation therapy, and chemotherapy, has led to improved survival in patients with head and neck cancers (HNCs) [3]. While many management options exist to help minimize the adverse effects of these treatments, they continue to have debilitating effects. Medical honey offers one of the many, albeit less recognized, management options for treatment-related adverse effects.

While honey gained recognition for its medicinal properties thousands of years ago, medical-grade honey products have entered the market over the last few decades [4,5]. To be considered medical-grade, honey must be radiated to remove bacterial spores and delivered sterile [4]. Manuka honey, derived from Leptospermum species, is the most frequently studied and utilized type of honey [6,7]. The qualities of medical honey include a low pH and high osmotic potential, which promote autolytic debridement of the wound while providing a moisture barrier to the environment [8,9]. Honey can produce hydrogen peroxide and contains multiple other molecules with antimicrobial properties [10]. In-vitro studies have shown that honey may stimulate pro-inflammatory cytokines, protect against the formation of bacterial biofilms, stimulate fibroblasts, and improve wound healing [10-13]. However, medical honey has not been widely utilized in head and neck surgery for wound care. Conversely, the medicinal properties of honey have been studied in patients undergoing radiotherapy for HNCs, yet many practitioners are not familiar with its utility in this group of patients. This article reviews the current evidence of using medical-grade honey for HNC treatment-related adverse effects including postoperative wound care and radiation-induced mucositis.

## Review

Methods

Ovid (Medicine) and PubMed (Medline) databases were searched using the boolean query "honey"(MeSH Terms) OR "honey" OR "honeys" OR "honey's" AND "head and neck neoplasms" (MeSH Terms) OR ("head" AND "neck" AND "neoplasms") OR "head and neck neoplasms" OR ("head" AND "neck" AND "cancer") OR "head and neck cancer." Publications related to postoperative wound healing and radiation-induced mucositis underwent different inclusion criteria due to the vast discrepancy between the volume of the two subjects' published literature. For publications on the treatment of postoperative wound healing, inclusion criteria consisted of randomized controlled trials evaluating postoperative wound healing from surgeries commonly performed to treat HNC. Mass removal and free or local flap reconstruction surgeries were considered eligible for review. Exclusion criteria consisted of isolated case reports, articles without a full-text manuscript available, and articles not published in English. After conducting the initial database search query and applying the inclusion/exclusion criteria, only one article qualified for review, and thus, an additional Google Scholar search was conducted using the key phrases "scar healing," "head and neck cancer," and "honey." After the search was sorted by relevance, the top 100 citations were identified and subjected to the inclusion/exclusion criteria. For publications on preventing radiation-induced mucositis, inclusion criteria consisted of meta-analyses that reported assessing trials for methodological quality and risk of bias. Exclusion criteria consisted of individual clinical trials, systematic reviews without a reported meta-analysis, and articles without a full-text manuscript available. After screening for duplicates, all abstracts were assessed according to the inclusion criteria, and full-text manuscripts were retrieved and assessed for eligibility. All randomized controlled trials included in the review underwent a bias risk assessment using the revised Cochrane risk of bias tool (RoB 2). The tool utilizes a question-based format with responses including "not available," "yes," "probably yes," "probably no," and "no" with a risk determination classified as "low risk," "some concerns," or "high risk" in five domains [14].

Results

**Figure 1:**
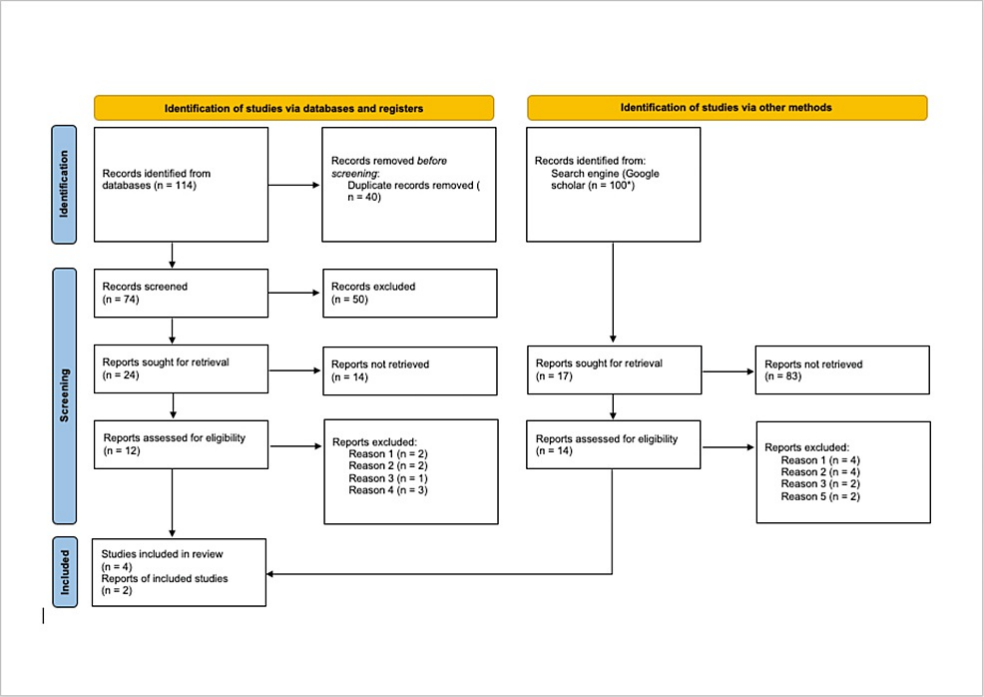
Flow sheet for the methodology of literature search *Only the first 100 citations from the Google Scholar search were screened Reason 1: Not in the head and neck; Reason 2: Not a malignancy-related treatment; Reason 3: Not in the English language; Reason 4: No methodological quality assessment or risk of bias; Reason 5: Full text not available

The original search query yielded 114 publications. After duplicate removal, 74 publications remained and underwent abstract screening. About 50 publications did not meet the inclusion criteria and were excluded and 24 publications underwent full-text manuscript assessment. For postoperative wound healing, one publication by Robson et al. met all inclusion criteria and was included in the literature review [15]. About 100 titles and abstracts from the Google Scholar search were screened for relevance, and 17 underwent full-text manuscript assessment. One randomized control trial by Thamboo et al. for patients undergoing a thyroidectomy met all inclusion criteria and was selected for review. The trial was conducted at a tertiary referral center for thyroid cancer, but the authors did not specify whether the patients enrolled in the study had thyroid cancer [17]. One trial by Alasqah et al. consisting of patients undergoing a palatal graft harvest met the criteria and was included in the review [16]. Publications related to wound healing that were excluded consisted of trials in anatomic regions other than the head and neck, trials of wounds unrelated to the treatment of HNC, and publications related to in-vitro and animal studies. Six meta-analyses assessing the use of honey to prevent radiation-induced mucositis were screened from the original search query. Three meta-analyses were excluded for not mentioning methodological quality or bias risk assessment of the trials included in their meta-analyses. Three meta-analyses by Co et al., Cho et al., and Tian et al., respectively, met all inclusion criteria and were reviewed [18-20] (Figure 1).

Wound healing

Robson et al. completed a randomized control trial of patients who underwent microvascular free tissue flap reconstruction for HNC. Randomization placed 49 patients into either postoperative wound care with medical grade manuka honey gel or conventional wound dressing. The wounds were swabbed for bacterial cultures in both groups, but there was no statistical significance in positive culture rates at any time point in the groups. The average length of hospital stays in the honey group was shorter than the conventional wound treatment group (16 vs. 21, respectively, p = 0.047). However, the authors did not assess whether the shorter hospital stays were related to wound healing. Questionnaires answered at the end of the trial revealed that 86% of the patients in the honey group reported being satisfied or very satisfied with the length of time it took the wound to heal, compared to 69% in the no-treatment group [15] (Table 1).

**Table 1:** Summary of the efficacy of medical honey for postoperative wound care in the head and neck VAS: Visual analog scale The risk of bias was determined by utilizing the Rob2 assessment tool. The final determinations are based on collective determinations in five domains and consist of "low," "some concern," or "high" risks of bias [14].

	Study group	Intervention vs control (number of patients)	Outcomes measured	Results	Risk of bias determination
Robson et al. [15]	Patients undergoing microvascular free tissue reconstruction in the maxillofacial unit	Manuka honey gel (25) vs conventional dressing (24)	Bacterial wound swabs	No statistical significance in positive culture rates (36% vs. 38%, p > 0.90)	Low
			Average length of hospital stay	16 vs. 21 days (p = 0.047)	
Alasqah et al. [16]	Patients undergoing a hard palate-free gingival graft harvest	Manuka honey gel (10) vs no dressing (10)	Size of donor site	86% healed in width and 91% healed in length vs. 14% and 9%, respectively	High (determined high in 2 of 5 domains)
			Patient pain via a visual analog scale (VAS)	VAS for the treatment group was significantly lower than the control group at all follow-up points (p = 0.001)	
Thamboo et al. [17]	Patients undergoing a thyroidectomy	Manuka honey gel (9) vs no dressing (12)	Subjective and objective scar appearance	Patients in the treatment group had less scar pigmentation at 8 weeks (p = 0.008). No statistical difference in treatment groups for vascularity, relief, thickness, or pliability of scar.	High (determined high in 2 of 5 domains)

Alasqah et al. evaluated palatal wound healing after harvesting a free gingival graft. Twenty patients were randomized to wound care with medical-grade manuka honey while the control group received no dressing material. The length and width of healing compared to the immediate postoperative defect were used to assess the speed of healing. At four weeks, the healing of the donor site in the medical honey group was 86% in width and 91% in length compared to 14% and 9% in the control group (p = 0.001). The honey group also reported experiencing significantly lower pain than the control group (p = 0.001) [16] (Table 1).

Thamboo et al. evaluated subjective and objective scar aesthetics by comparing the daily application of medical-grade manuka honey to the incision site of patients who underwent a thyroidectomy. The control group used no topical agents or creams. An observer scar assessment scale was used at postoperative weeks four and eight. Patients who used manuka honey had reduced pigmentation levels (p = 0.008) compared to the control group. No differences were noted in vascularity, relief of surface irregularities, thickness, and pliability between the two groups according to the Patient and Observer Scar Assessment Scale [17] (Table 1).

Radiation-induced mucositis

Co et al. conducted a meta-analysis to assess the use of honey on radiation-induced oral mucositis. The treatment groups were given honey orally, with most trials prescribing twenty mL of honey to be swished and swallowed three times daily. The control group treatments included either a normal saline solution and standard of care or a syrup-based placebo gel. Although there was no statistical difference in the development of severe mucositis, risk ratio (RR) of 0.45 (CI = 0.09-2.21), there was a significantly lower risk of radiation treatment interruption in the honey group with an RR of 0.11 (CI = 0.02-0.5) [18] (Table 2).

**Table 2:** Summary of the efficacy of oral honey solutions in patients undergoing radiotherapy of the head and neck RR: Relative risk; CI: Confidence interval; logOR: Natural logarithm of the odds ratio; SMD: Standardized mean difference

	Number of studies and subjects	Treatments	Controls	Outcomes	Pooled results	Risk of bias/methodological quality assessment
Co et al. [18]	5 (244)	Honey orally swished and swallowed (20 mL three times daily in four of five studies)	Normal saline, syrup-based placebo	Treatment interruptions	Lower risk of having treatment interruptions in the honey group (RR = 0.11, CI = 0.02-0.58)	Critical Appraisal Skill Programme (CASP)
				Peak mucositis score	No statistical difference in risk of developing severe mucositis (RR = 0.45, CI = 0.09-2.21)	
Cho et al. [19]	9 (476)	Orally administered honey	Sterile water, Tantum, placebo syrup	Incidence of moderate to severe mucositis	Significantly lower incidence in the honey group vs. control group for patients undergoing radiotherapy (logOR = -2.87, p < 0.0001)	Cochrane risk of bias assessment tool
				Onset time to mucositis	Significantly later onset of mucositis in the treatment group (SMD = 2.64, p = 0.0036)	
				Weight loss	Incidence of weight loss was significantly lower in the honey group (logOR = -1.94; p < 0.001)	
Tian et al. [20]	7 (412)	Orally administered honey plus standard of care	Normal saline, placebo gel, lignocaine gel	Incidence of oral mucositis	No statistical difference in incidence between the honey group and placebo groups (RR = 0.22, CI = 0.4-1.18)	Cochrane risk of bias assessment tool
				Severity of oral mucositis	Lower risk of developing severe oral mucositis RR = 0.22, CI = 0.13-0.38)	
				Weight maintenance	Honey group maintained or increased weight (RR = 1.92, CI = 1.33-2.77, p < 0.001)	
				Treatment interruptions	Reduced treatment interruptions in honey group (RR = 0.13, CI = 0.02-0.97, p = 0.05)	

In another meta-analysis study, Cho et al. studied the effect of honey on treatment-related mucositis. About 476 patients were included in the meta-analysis. The treatment group received oral honey while undergoing radiotherapy or chemoradiotherapy, and the control group received various oral rinses such as water, lignocaine, and golden syrup. They found a statistically significant decrease in the incidence of moderate to severe mucositis in the honey group for patients undergoing radiotherapy (logOR = -2.87, p < 0.0001). In addition, they appreciated a significantly later onset of mucositis in the honey group (SMD = 2.64, p = 0.0036) [19] (Table 2).

Tian et al. included seven trials with 412 patients in their meta-analysis. The treatment groups received various types of honey while the comparison groups received normal saline or lignocaine gel. Their results showed that honey did not decrease the incidence of radiation-induced oral mucositis relative risk (RR = 0.22, CI = 0.4-1.18, p = 0.18) but did reduce the severity of oral mucositis (RR = 0.22; CI = 0.13-0.38, p < 0.001). Honey treatment also helped in maintaining or increasing patient weight (RR = 1.92, CI = 1.33-2.77, p < 0.001) and reduced treatment interruptions related to oral mucositis (RR = 0.13; CI = 0.02-0.91; p = 0.05) [20] (Table 2).

Discussion

Current evidence for using medical honey in postoperative wound care for HNC consists of a few trials, all of which enrolled a small number of participants. While Thamboo et al. showed a significant decrease in scar pigmentation, no other aesthetic metrics significantly differed between the honey treatment and control groups [17]. Notably, a disproportionate number of patients in the honey treatment group dropped out and were unavailable for scar assessment for the entire eight weeks of the study, thus introducing a high risk of bias. The authors reported that some of these patients complained about the messiness, with honey getting on their clothing after application to the anterior neck, and no adverse events in the treatment arm occurred [17]. While the study conducted by Robson et al. revealed a statistically significant shorter hospital stay for the wound-dressed honey group, no evidence was assessed to attribute the shorter hospital stays to faster wound healing times. The effect of honey on wound healing rates would need to be further investigated by more rigorous trials with a more extended follow-up period. In-vitro studies have revealed the antimicrobial properties of honey [10-13], but this trial failed to reveal a statistically noticeable difference in wound bacterial culture rates [15]. Alasqah et al. determined that patients receiving medical honey for palatal grafts had very favorable effects on healing rates [16], but a lack of information on the randomization process and data collection deemed this trial a high risk for bias. Despite the small sample size and bias risk, the results of this study and the favorable effects honey may have on radiotherapy-induced mucositis prevention suggest that honey may play a role in protecting and maintaining healthy mucosal surfaces.

Corporations have manufactured medical honey into gels and sheets, which function similarly to other wound care treatments by providing a moist and mechanical barrier to the environment and preventing discomfort from drying and cracking. In addition, in-vitro studies suggest that the intrinsic properties of honey may provide an additional antimicrobial effect and stimulate wound healing [10-13], although these properties have largely yet to be clinically significant in human wound studies. Evidence also shows honey does not confer bacterial resistance due to its multimodal antimicrobial activity [10]. However, existing literature has yet to assess the efficacy of medical honey versus other modern wound care products, such as antibacterial ointments and gauze impregnated with antimicrobial elements. Many of the authors reviewed here reported cost-effectiveness and easy access to honey as strengths of its utility [15,16,18].

While few studies exist assessing the efficacy of honey for wound healing in head and neck surgery, many trials have been conducted to determine its utility in other anatomical regions, often with mixed results. For example, one study assessed manuka honey versus saline-soaked gauze for the treatment of diabetic foot ulcers. The ulcers treated with manuka honey showed a significant acceleration of healing and disinfection rates in diabetic foot ulcers compared to saline-soaked gauze used in the control group (Mean healing duration was 31 +/- 4 days in the manuka honey group versus 43 +/- 3 days in the control group, p < 0.05. Within one week, 78.13% of patients in the honey group presented with sterile wounds versus 35.5% in the control group) [21]. Conversely, another randomized controlled trial with 368 participants found no significant healing times in venous leg ulcers compared to usual care with other impregnated gauze and antimicrobial agents [22]. Many studies assess the efficacy of honey in the management of burn wounds. One systematic review of seven randomized control trials on using honey in superficial burn wounds showed that honey treatment led to significantly shorter healing times and infection resolution. The control groups used unconventional treatments such as potato and amniotic membranes in these trials [23]. Two of these trials compared honey to silver sulfadiazine, a conventional treatment for burns, with honey showing a significant advantage in healing times and infection control [24,25]. With a limited amount of evidence for the utility of honey in head and neck wounds, more robust studies in other anatomical regions should encourage further examination of honey in the treatment of HNC. Trials comparing medical honey versus other modern wound care agents and their effect on grafting success, wound infection rates, and healing times should be assessed, given the proven safety and potential benefit honey may provide for these purposes.

Radiotherapy continues to be a critical treatment of HNC, with oral/oropharyngeal mucositis being a typical dose-limiting adverse effect and a key component in treatment failures [18]. Current preventative treatments, such as optimal oral hygiene and various combinations of mouth rinses, have limited efficacy. Currently, standard preventative treatments for radiation-induced mucositis do not include honey [26]. Orally administered honey, whether swallowed or swished, coats the mucosal surfaces and may protect from radiation-induced mucosal damage. The articles reviewed provide evidence that honey can delay the progression of moderate/severe mucositis and decrease treatment interrupting adverse effects. Honey provides a safe and inexpensive option to supplement other pharmaceutical interventions. Additionally, independent of mucositis prevention, honey can provide supplemental ingestion of calories to a patient demographic who routinely struggles to maintain oral intake and weight. Providers of Western medicine do not frequently prescribe or inform patients undergoing radiotherapy of the potential benefits of honey, and thus more awareness of the stated evidence is needed.

## Conclusions

In summary of the existing evidence, honey provides a safe complementary treatment option for the prevention of radiation-induced mucositis for those with HNC. Medical-grade honey can be used on postoperative wounds as a moisture barrier and may facilitate healing and improvements in scarring. No evidence exists comparing honey to other common postoperative wound ointments in the region of the head and neck, and more evidence is needed to quantify the efficacy of honey in postoperative wounds and grafts.
